# Epidural Catheters for Cervical Epidural Steroid Injections to Target Higher Cervical Pathology: Clinical Images in Practice

**DOI:** 10.7759/cureus.48528

**Published:** 2023-11-08

**Authors:** Peter D Vu, Christopher L Robinson, Grant H Chen, Jamal J Hasoon

**Affiliations:** 1 Department of Physical Medicine and Rehabilitation, University of Texas Health Science Center at Houston, Houston, USA; 2 Department of Anesthesiology, Critical Care, and Pain Medicine, Beth Israel Deaconess Medical Center, Harvard Medical School, Boston, USA; 3 Department of Anesthesia and Pain Medicine, University of Texas Health Science Center at Houston, Houston, USA

**Keywords:** interlaminar approach, educational images, epidural catheter, steroid epidural injections, cervical radicular pain

## Abstract

Cervical radicular pain is commonly treated with cervical epidural steroid injections. The transforaminal approach allows for direct treatment of the steroid at a particular nerve root or level. Still, it carries a significant risk of morbidity and mortality with thromboembolism or injury to cervical vasculature. The interlaminar approach is commonly utilized as it avoids vascular structures. However, the epidural space becomes narrower at higher levels, limiting the ability to perform this approach at higher cervical levels. Cervical epidural catheters can be used and advanced to target higher cervical pathology through the interlaminar approach. We present clinical images demonstrating the utility of a cervical catheter for treating higher cervical levels.

## Introduction

Cervical radicular pain is commonly treated with cervical epidural steroid injections [1,2]. The transforaminal approach allows for direct treatment of the steroid at a particular nerve root or level. Still, it carries a significant risk of morbidity and mortality with thromboembolism or injury to cervical vasculature [2]. The interlaminar approach is commonly utilized as it avoids vascular structures. However, the epidural space becomes narrower at higher levels, limiting the ability to perform this approach at higher cervical levels [1,3]. Cervical epidural catheters can be used and advanced to target higher cervical pathology through the interlaminar approach. Novel studies report microcatheters targeting cervical pathology but do not provide technique visualization [1]. We provide interlaminar cervical epidural steroid injection visualization at the C7/T1 interspace with microcatheter advancement to treat C3/C4 radicular pain.

## Case presentation

We present a female teacher in her early 50s with a history of hypertension, hyperlipidemia, and previous C5/C6 cervical fusion five years prior who presents with a new onset of headaches and neck pain with radicular, electric-like pain radiating into her bilateral shoulders and upper arm for the last four months. She denies any new trauma or inciting factors. Her pain is considered a constant 7-8/10 that is exacerbated to 9-10/10 with neck flexion and extension. Prior to this new pain, she was active with daily walks and weekly tennis matches. She also reported difficulty in neck range of motion, especially with straining her neck to teach effectively with school equipment. This included extending and bending her neck to use the chalkboard, lifting school supplies from shelves, and monitoring the children. Before her referral to the pain clinic by her primary care provider, the patient reported her previously prescribed gabapentin 300 mg three times a day and over-the-counter acetaminophen 1,000 mg three times a day had not been helpful. Her primary care provider had also obtained further cervical spine radiographs, which reported no changes to her anatomical features or previous cervical fusion. Before the referral, physical therapy and cervical spine magnetic resonance imaging (MRI) were ordered by the primary care provider.

**Figure 1:**
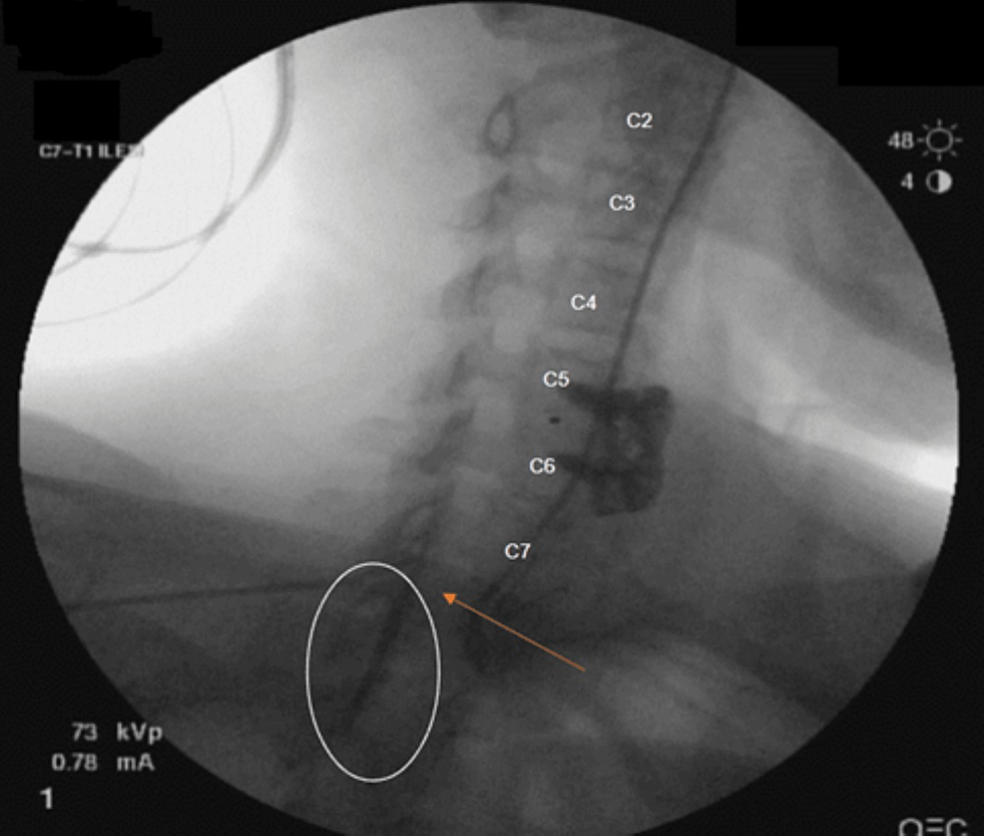
Contralateral oblique view of an interlaminar cervical epidural steroid injection at the C7/T1 interspace (arrow) demonstrating minimal cephalic spread of contrast (white circle).

At the pain clinic, the cervical spine MRI was reviewed and showed new-onset disc bulges at C3/C4 and C4/C5. After further discussion with the patient, she opted to trial an intralaminar cervical epidural steroid injection at the C7/T1 interspace in the clinic procedure room. The patient was placed in a prone position, and the target area was cleaned with chlorhexidine and alcohol wipes. Lidocaine (5 mm) was injected to provide local anesthetic into the target area before a spine needle was introduced. A contralateral oblique image was utilized for safety and reliability [4,5]. After obtaining a loss of resistance with approximately 3 cm of needle insertion, iohexol contrast without additives was injected and demonstrated minimal cephalic spread (Figure 1).

**Figure 2:**
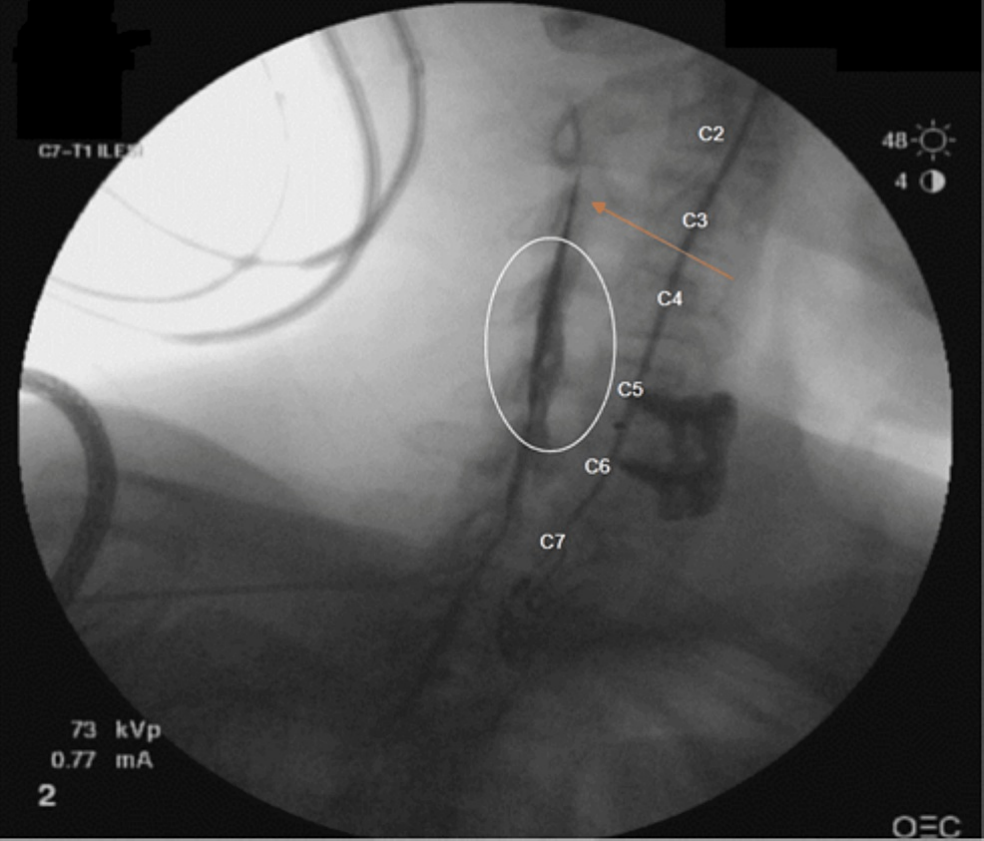
Contralateral oblique view after advancement of a microcatheter at C3 (orange arrow) demonstrating significantly more contrast spread around the C3/C4, C4/C5, and C5/C6 levels (white circle).

Given that the patient’s pathology was more cephalic, a microcatheter (Perifix Epidural Catheter (20 Ga), Braun B, Melsungen, Germany) was advanced through the Tuohy needle to the C3/C4 level with approximately 5 cm of microcatheter. Contrast was again injected and demonstrated diffuse spread around the C3/C4 and C4/C5 levels (Figure 2).

Once confirmation of the level was obtained, 3 mL of dexamethasone and bupivacaine (1:4 ratio) mixture were injected, which the patient tolerated well. The catheter needle was immediately removed, and the patient was observed for 10 minutes in the clinic procedure room. She reported 50% relief immediately after the procedure and was discharged home from the clinic. At her three-month follow-up, the patient reported 90% relief of radicular symptoms (at worst a 1/10) and improved teaching involvement with her students.

## Discussion

Cervical epidural steroid injections are medical procedures commonly used to alleviate pain and inflammation in the neck and upper spine region [1]. These injections involve the administration of a local anesthetic (i.e., lidocaine, bupivacaine, or ropivacaine) and a corticosteroid (i.e., particulate (triamcinolone and methylprednisolone) and nonparticulate (dexamethasone and betamethasone)) medication into the epidural space surrounding the spinal cord and nerve roots. There are two primary approaches for administering cervical epidural steroid injections: the transforaminal approach and the interlaminar approach [2]. The transforaminal approach involves injecting the medication directly into the neural foramen, a narrow opening through which spinal nerves exit the spinal cord. This method specifically targets the affected nerve root and provides localized relief. On the other hand, the interlaminar approach involves injecting the medication into the epidural space between the vertebrae. While the interlaminar approach covers a broader area, it may disperse the medication less precisely.

Safety considerations between the two approaches revolve around the risk of complications [3]. The transforaminal approach is more targeted but may carry a slightly higher risk of neurovascular injury, while the interlaminar approach is generally considered safer, but it might require higher medication doses to achieve the desired effect. Epidural catheters, as in this case, can be employed to treat conditions that are challenging to access directly through injections [5,6]. Currently, a variety of microcatheters are commercially available for catheterizing intricate brain arteries. These microcatheters exhibit the necessary flexibility to navigate through remote vascular locations and the strength required for propelling them effectively. Furthermore, these catheters can be used off-label to thread into the epidural space using readily available live fluoroscopic equipment [7].

Despite the benefits, the use of epidural catheters involves careful monitoring and carries specific risks; considerations of neurovascular structures and the risk of epidural hematomas, as described by Palmer [8], should be made prior to pursuing this technique. The use of fluoroscopy and loss-of-resistance techniques minimizes the risk of direct spinal cord contact and damage to the spinal cord. Further consideration includes particulate versus nonparticulate steroids; in the case of cervical epidural steroid injections, nonparticulate steroids are safer and reduce the risk of ischemic damage to the cord that could perpetuate diaphragmatic weakness or paralysis [9].

## Conclusions

These clinical images demonstrate the utilization of a catheter to target higher cervical pathology when using the interlaminar approach for cervical epidural steroid injection. Consideration of risks between transforaminal, interlaminar, and catheter approaches should be paramount when pursuing cervical epidural steroid injections.
